# Identification of Sex-Specific Plasma Biomarkers Using Metabolomics for Major Depressive Disorder in Children and Adolescents

**DOI:** 10.3389/fpsyt.2022.929207

**Published:** 2022-07-14

**Authors:** Yuanliang Jiang, Mengchang Qin, Teng Teng, Xuemei Li, Ying Yu, Jie Wang, Hongyan Wu, Yuqian He, Xinyu Zhou, Peng Xie

**Affiliations:** ^1^Department of Psychiatry, The First Affiliated Hospital of Chongqing Medical University, Chongqing, China; ^2^National Health Commission (NHC) Key Laboratory of Diagnosis and Treatment on Brain Functional Diseases, The First Affiliated Hospital of Chongqing Medical University, Chongqing, China; ^3^Department of Neurology, The First Affiliated Hospital of Chongqing Medical University, Chongqing, China

**Keywords:** major depressive disorder, children and adolescents, sex specific, biomarker, metabolomics, plasma

## Abstract

**Background:**

Children and adolescents are at a high risk of major depressive disorder (MDD) with known sex differences in epidemiology. However, there are currently no objective laboratory-based sex-specific biomarkers available to support the diagnoses of male and female patients with MDD.

**Methods:**

Here, a male set of 42 cases and 27 healthy controls (HCs) and a female set of 42 cases and 22 HCs were recruited. This study investigated the sex differences of plasma metabolite biomarkers in young patients with MDD by the application of ultra-high-performance liquid chromatography equipped with quadrupole time-of-flight mass spectrometry.

**Results:**

The metabolic profiles showed clear separations in both male and female sets. In total, this study identified 57 male-related and 53 female-related differential metabolites. Compared with HCs, both male and female subjects with MDD displayed four significantly altered pathways. Notably, biliverdin was selected as an independent diagnostic male-specific biomarker with an area under the receiver operating characteristic curve of 0.966, and phosphatidylcholine (10:0/14:1) was selected as a female-specific biomarker, achieving an area under the curve (AUC) of 0.957.

**Conclusion:**

This metabolomics study may aid in the development of a plasma-based test for the diagnosis of male and female children and adolescents with MDD, as well as give new insight into the pathophysiology of sex differences in children and adolescents with MDD.

## Introduction

Major depressive disorder (MDD) is a common mental health problem worldwide, with 322 million people suffering from depression, and an estimated prevalence of 3.2% from 3 to 17 years of age (1, 2). Globally, MDD has become the top cause of illness and disability during adolescence, and more than half of adolescent suicide victims exhibit MDD at the time of death (3, 4). The sex difference in depression rates begins in childhood and persists into old age, with females having a strong prevalence for MDD compared with males (approximately 2:1), and this gender gap becomes smaller in adults compared with younger ages (5, 6). Notably, the sex difference peaks in adolescence (odds ratio = 3.02 for ages 13–15 years, and uncorrected effect size = 0.47 for 16 years of age) in depression (7). Several hypotheses have attempted to explain this phenomenon. Research indicates that increases in sex steroids are linked to organizational effects on neural structure and function (8). A high level of testosterone was associated with higher levels of adolescent depression in girls (9). One circuit, which has high concentrations of sex steroid receptors, connects the amygdala to the hippocampus and ventral expanses of the prefrontal cortex. This circuit is linked to hypothalamic-pituitary-adrenal axis activity and may provide a biological mechanism for why girls have higher risk of depression than boys (10). However, these hypotheses need to be tested and verified by research. According to previous studies, metabolism differences between boys and girls with MDD, like sex steroids, may affect neural function or the activity of the hypothalamic-pituitary-adrenal axis, which has a different impact on the epidemiology, symptomatology, and pathogenesis of depression (11). Therefore, sex-specific molecular characteristics are required to clarify the sex differences in MDD.

Metabolomics, which identifies and quantifies metabolites in various biological fluids such as plasma and urine, has been extensively applied to determine the metabolic perturbations in various disease states (12). In earlier studies, metabolomics was successfully used to identify novel biomarkers for neuropsychiatric disorders, such as schizophrenia and bipolar disorder (13, 14). Previously, we identified some candidate diagnostic metabolite biomarkers for MDD in adults, as well as in children and adolescents (15, 16), and analyzed sex-specific metabolites of MDD in adults (17). Other researchers also found blood metabolomic signatures of major depression before and after antidepressant treatment (18). However, none of these studies have taken sex differences of MDD in children and adolescents into account, which may limit the universal applicability of these potential metabolite biomarkers to some extent. In our previous study, adults with MDD had disruptions in methionine and tryptophan metabolism, and tryptophan was identified as a key biomarker for discriminating adult MDD (16). Children and adolescents with MDD had substantial purine metabolism disturbances, and inosine was selected as an independent diagnostic biomarker. Past studies reported that MDD in children and adolescents was different from adults in pathophysiology, treatment response and neuroimaging (19–21). Thus, it is likely that sex differences are prominent for MDD in children and adolescents and may provide an understanding of the mechanisms underlying the pathoetiology in both males and females.

In this investigation, ultra-high-performance liquid chromatography equipped with quadrupole time-of-flight mass spectrometry (UPLC-Q-TOF/MS) analysis was applied to profile 84 subjects with MDD (42 males and 42 females) and 49 healthy controls (HCs, 27 males and 22 females) in children and adolescents with the aim of identifying sex differences in plasma metabolic signatures, and obtain altered metabolic pathways would help to understand the differences in mechanisms between boys and girls. We also explored sex-specific metabolite biomarkers in males and females with MDD separately, which could contribute to future clinical practice for more efficient diagnosis of depression in different populations.

## Materials and Methods

### Ethics Statement

The samples obtained from these subjects had been previously used to explore potential biomarkers of depression in children and adolescents with MDD (16). Ethics approval was obtained from the Institutional Review Board of Children’s Hospital of Chongqing Medical University (No. 2016121). Prior to the collection of plasma samples, written informed consent and detailed clinical information were obtained from participants (if they were 18 years old) or legal guardians (if aged 17 years or less). All procedures were conducted according to the principles expressed in the Declaration of Helsinki (22).

### Subject Recruitment

For this study, HCs and patients with MDD were recruited from routine medical examinations in two schools, a primary school in Yuzhong district and a middle school in Liangping district, both in Chongqing, China, and the Children’s Hospital of Chongqing Medical University (Chongqing, China) between November 2016 and May 2017. The diagnosis of MDD was confirmed by trained psychiatrists according to criteria in the Diagnostic and Statistical Manual of Mental Disorders-version 4 (DSM-IV). The severity of depression was assessed by two standardized and validated depression scales, i.e., Children’s Depression Rating Scale-Revised (CDRS-R) for children and younger adolescents (aged < 15 years) (23) and Hamilton Depression Scale (17-Items) (HAMD-17) for older adolescents (aged ≥ 15 years) (24). Patients aged from 6 to 18 years with moderate to severe depression (i.e., CDRS-R scores ≥ 40 or HAMD-17 scores ≥ 17), without any comorbid physical, neurological or psychiatric disorders and/or illicit drug use, were included in the study. Both first-episode drug-naive patients with MDD (DN-MDD) and drug-treated patients with MDD (DT-MDD) were recruited. For HCs, subjects with no previous neurological, DSM-IV Axis I/II or medical illness were also recruited.

### Sample Collection and Preparation

The fasting blood of subjects was taken in 5 mL vacutainer tubes containing heparin lithium, then centrifuged for 15 min (1,500 × g, 4°C). Plasma samples were stored at –80°C in equal aliquots (150 μL) until later analysis. Before UPLC-Q-TOF/MS analysis, 100 μL aliquots of thawed plasma samples were mixed with 400 μL of cold methanol/acetonitrile (1:1, v/v) for protein removal. The mixture was centrifuged for 15 min (14,000 × g, 4°C) and the supernatant dried in a vacuum centrifuge. The samples were redissolved in 100 μL acetonitrile/water (1:1, v/v) solvent. To monitor the stability and repeatability of instrumental analysis, 10 μL of each sample was combined into quality control (QC) samples. The QC samples were inserted regularly and analyzed in every eight samples.

### Ultra-High-Performance Liquid Chromatography Equipped With Quadrupole Time-of-Flight Mass Spectrometry Analysis

An Agilent 1290 Infinity LC system (Agilent Technologies, Santa Clara, California, United States) coupled with an AB SCIEX Triple TOF 6600 system (AB SCIEX, Framingham, MA, United States) was used to undertake metabolic profiling of plasma samples. ACQUITY HSS T3 1.8 μm (2.1 × 100 mm) columns were used for chromatographic separation in both positive and negative models. The column temperature was maintained at 25°C. The mobile phases of 0.1% formic acid in water (A) and 0.1% formic acid in acetonitrile (B) were used in the positive ionization mode, while 0.5 mM ammonium fluoride in water (C) and acetonitrile (D) were used in the negative ionization mode. In the positive model, the elution gradient initially started from 1% B and increased linearly to 100% B between 1 and 8 min, and was maintained for 2 min, then returned to 1% B for 2 min to equilibrate the systems. This elution gradient was replicated in the negative model using reagents C and D. The sample injection volume was 2 μL and the flow rate was set to 300 μL/min.

The mass spectrometric data were collected using the UPLC system equipped with an electrospray ionization source operating in either positive or negative ion mode. The optimal conditions were set as follows: ion source gas 1, 40 psi; ion source gas 2, 60 psi; curtain gas, 30 psi; ion-spray voltage floating, 5,000 V in positive mode and –4,500 V in negative mode, respectively, normalized collision energy, 20–40–60 V rolling for MS/MS. Data acquisition was performed with Information-Dependent Acquisition (IDA) mode, and parameters of IDA included a declustering potential [60 V (+) and –60 V (–)], collision energy [50 V (+) and –20 V (–)], exclusion of isotopes within 4 Da, and 10 candidate ions to monitor per cycle.

### Metabolomics Data Analysis

After non-targeted UPLC-Q-TOF/MS analyses, the raw data were converted to mzXML files using the Proteo Wizard MSconventer tool and then processed using XCMS online software. The parameters in XCMS were set as follows: centWave settings for feature detection [Δm/z = 25 ppm, peakwidth = c (10, 60)]; obiwarp settings for retention time correction (profStep = 1); and parameters including minfrac = 0.5, bw = 5 and mzwid = 0.025 for chromatogram alignment. Normalized and integrated data were then uploaded into MetaboAnalyst 5.0 software for further processing.^1^ Principal component analysis (PCA) using an unsupervised method was applied to obtain an overview of the metabolic data, general clustering, trends, or outliers. Orthogonal partial least squares discriminate analysis (OPLS-DA) was used for statistical analysis to determine global metabolic changes between comparable groups. Two matrices were involved in the OPLS-DA analysis: the X matrix was the sample-variable observation matrix, and the Y matrix was the sample category attribution matrix. R2X and R2Y represented the interpretation rate of the X and Y matrices by the model, and Q2 represented the predictive ability of the model. If both R2Y and Q2 were greater than 0.5, the model could predict well and avoided the risk of over-fitting. Variable importance in the projection (VIP) were calculated in the OPLS-DA model. *P*-values were estimated using Student’s *t*-test (subject to normal distribution) or Mann-Whitney *U*-test (not subject to normal distribution). Finally, metabolites with a VIP value > 1.0 and *P* < 0.05 were identified as metabolites exhibiting different levels and responsible for sample differentiation. Then, we analyzed the correlations between metabolites and clinical characteristics. Because only patients with MDD were assessed with CDRS-R or HAMD-17, correlations between biomarkers and depression scales scores were done in patients only. To facilitate uniform comparison, we applied the COD-FISH Tool to transform all HAMD-17 scores to CDRS-R scores (25).

### Statistical Analysis

Continuous variables were presented as mean with standard deviation. As appropriate, comparisons of demographic characteristics between groups were performed on SPSS 21.0 (IBM Corp., Armonk, NY, United States) using the non-parametric Mann-Whitney U test. Normal distribution was evaluated by Kolmogorov-Smirnov test. Spearman’s correlation was applied to analyze the correlations between metabolites and CDRS-R scores. Random forest analysis was used to select the best-fit model. Receiver operating characteristic analysis was applied to determine the diagnostic performance of the best-fit model. A *P*-value < 0.05 was considered to be statistically significant.

## Results

### Clinical Characteristics for the Metabolomic Model of Major Depressive Disorder vs. Healthy Controls

The data from our previous published study was reanalyzed for this study (16). One female healthy control was omitted from the research because of the lack of metabolome data. The demographic and clinical characteristics of all the study participants are summarized in Table 1. A total of 133 participants were enrolled in the current study as children and adolescents. The male set consisted of 27 patients with first-episode DN-MDD, 15 patients with DT-MDD and 27 HCs. Among male patients with MDD, the average HAMD-17 score was 22.00, and the average CDRS-R score was 45.00. In the female set, 25 patients with DN-MDD, 17 patients with DT-MDD and 22 HCs were recruited. The average HAMD-17 score in female patients with MDD was 22.10, and the average CDRS-R score was 52.69. Between the male and female sets, there were no significant differences in the demographic variables of age and body mass index. The number of participants with MDD and on medication did not differ significantly across the male and female sets.

**TABLE 1:** Clinical characteristics of the recruited patients with major depressive disorder and healthy controls.

	Male set			Female set		
		
	MDD	HCs	*P*-value^a^	MDD	HCs	*P*-value^a^
Sample size (*n*)	42	27	–	42	22	–
Age (years)						
Range	9–18	7–18	–	9–18	11–18	–
Mean ± *SD*	15.43 ± 2.58	14.93 ± 2.91	0.386	16.21 ± 2.07	16.27 ± 1.80	0.508
BMI (kg/m^2^)						
Mean ± *SD*	19.57 ± 2.33	19.69 ± 1.49	0.210	20.42 ± 4.17	20.03 ± 1.63	0.899
Depression symptoms severity						
HAMD-17 (Mean ± *SD*, *n*)	22.00 ± 3.10, 27	NA	–	22.10 ± 3.73, 29	NA	–
CDRS-R (Mean ± *SD*, *n*)	45.00 ± 5.66, 15	NA	–	52.69 ± 6.92, 13	NA	–
Antidepressant treatment (*n*)	15	NA	–	17	NA	–

*MDD, major depressive disorder; HCs, health controls; SD, standard deviation; BMI, body mass index; HAMD-17, Hamilton Depression Scale (17-Items); CDRS-R, children’s depression rating scale-revised.*

*^a^Mann-Whitney U-test continuous variables (age and BMI).*

### Metabolomic Analysis of Plasma From Patients With Major Depressive Disorder and Healthy Controls

**FIGURE 1:**
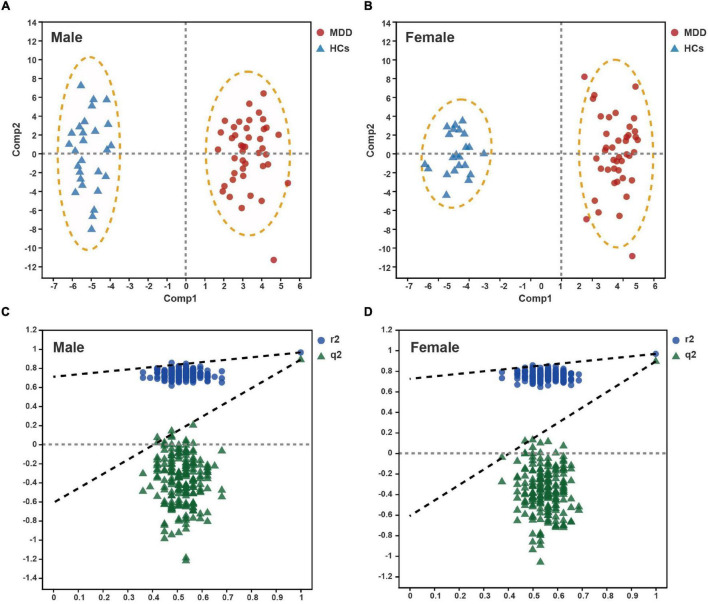
Metabolomic analysis of plasma from male and female patients with major depressive disorder (MDD) vs. healthy controls (HCs). Orthogonal partial least squares discriminate analysis (OPLS-DA) score plots displayed clear discrimination between male **(A)** and female **(B)** subjects with MDD from their respective HCs. Permutation test for male **(C)** and female **(D)** OPLS-DA models.

There were 5,353 positive-mode features and 1,680 negative-mode features identified in the metabolic profiling. In the PCA and OPLS-DA analyses, all metabolites of the subjects were utilized. Both male and female patients with MDD were distinctly separated from their respective HCs in PCA score plots, indicating satisfactory reproducibility (Supplementary Figure 1). OPLS-DA score plots demonstrated a striking distinction between male MDD and HC subjects, as well as female MDD and HC subjects [Figure 1A; male: R2X cumulative (cum) = 0.224, R2Y cum = 0.965, Q2 cum = 0.889; Figure 1B; female: R2X cum = 0.296, R2Y cum = 0.967, Q2 cum = 0.894]. In addition, the permutation test revealed that the two OPLS-DA models developed were both valid and were not over-fitted (Figures 1C,D). The metabolites responsible for sample differentiation were identified as those having VIP values of at least 1 and a *P*-value of less than 0.05. In total, by analyzing the OPLS-DA loading coefficient plot, we identified 57 differential metabolites in the male set and 53 in the female set (Table 2). Among them, 40 metabolites were significantly altered in both males and females with MDD. The results of Spearman’s correlation analysis between metabolites and CDRS-R scores were shown in Supplementary Table 1.

**TABLE 2:** Identified differential metabolites in both male and female sets.

Metabolites	Male set			Female set		
		
	*P*-value^a^	VIP	FC	*P*-value^a^	VIP	FC
PC (16:1/22:6)				2.61E-03	1.34	0.63
PA (22:5/0:0)	1.23E-03	1.45	0.79			
PE (22:4/0:0)	3.47E-03	1.31	0.85			
Adenosine				2.41E-10	1.53	0.07
PC (20:4/0:0)	1.34E-03	1.41	0.78	7.84E-05	1.56	0.73
PC (22:6/0:0)				2.09E-10	2.27	0.59
Biliverdin	8.48E-11	2.94	0.46	7.42E-09	2.37	0.44
L-Palmitoylcarnitine	5.05E-04	1.49	0.77	1.62E-05	1.83	0.61
3-Methylthiopropionate	4.37E-04	1.37	0.69	8.56E-03	1.13	0.60
Stearoylcarnitine	2.02E-03	1.39	0.74			
Carnitine (18:1)	1.71E-06	2.05	0.70	4.95E-07	1.90	0.67
L-Phenylalanine	1.49E-02	1.14	0.90			
L-Tryptophan	7.55E-04	1.43	0.70			
DL-O-tyrosine	9.36E-05	1.35	0.70			
PC (22:5/0:0)	8.58E-04	1.32	0.68	1.95E-02	1.02	0.80
PC (20:3/0:0)	3.84E-04	1.42	0.82			
Acetylcarnitine	2.81E-06	2.11	0.70	7.82E-08	2.09	0.47
PC (12:0/22:5)	9.06E-06	2.69	0.03	1.73E-09	2.72	0.02
PC (20:5/24:4)	5.08E-06	2.61	0.05	7.42E-09	2.68	0.03
PC (10:0/14:1)	9.58E-06	2.72	0.03	2.45E-09	2.79	0.02
Bilirubin	5.02E-06	1.96	0.71	7.81E-10	2.51	0.52
1-Methyladenosine	1.95E-03	1.41	0.73	1.16E-06	1.76	0.59
Linoleic acid	1.13E-04	1.70	0.51	1.16E-06	1.99	0.38
Alpha-linolenic acid	1.88E-05	1.87	0.49	4.18E-07	1.99	0.45
Cis-9-palmitoleic acid	1.68E-04	1.37	0.56	2.36E-08	2.22	0.34
Trans-vaccenic acid	5.26E-04	1.47	0.62	1.90E-06	1.97	0.42
Palmitic acid	1.99E-04	1.48	0.50	6.85E-07	1.97	0.35
Arachidonic acid	5.82E-03	1.34	0.68	1.08E-04	1.44	0.60
Eicosapentaenoic acid	3.43E-03	1.18	0.55	2.62E-04	1.39	0.54
Capric acid				2.23E-05	1.59	0.47
Carnitine (10:0)				1.56E-05	1.71	0.50
Dodecanoic acid	3.77E-02	1.23	0.55	2.03E-06	1.91	0.32
PC (16:0/18:2)	1.14E-05	1.90	0.63	2.16E-03	1.24	0.72
L-Methionine	2.12E-04	1.57	0.74	8.54E-04	1.01	1.22
Indole	1.48E-02	1.09	0.89			
Indoleacrylic acid	1.12E-02	1.11	0.86			
Hypoxanthine	2.73E-08	1.91	0.32	2.07E-07	1.58	0.24
Inosine	3.60E-10	2.31	0.13	4.99E-10	2.11	0.05
Dopamine	4.34E-03	1.19	0.79			
Betaine	2.26E-04	1.40	0.73	6.09E-06	1.79	0.60
L-Carnitine	1.32E-05	1.98	0.74	8.77E-04	1.08	0.74
Creatine				2.69E-05	1.22	0.49
D-proline	4.17E-03	1.50	0.59	3.06E-02	1.36	0.56
Creatinine	1.35E-06	2.03	0.42	3.53E-08	2.05	0.30
L-pyroglutamic acid	1.19E-04	1.79	0.55	6.51E-06	1.76	0.48
PG (16:0/0:0)	1.95E-02	1.02	0.79			
PC (13:0/22:2)	2.49E-03	1.31	13.40			
PC (18:2/24:4)				2.06E-03	1.01	2.92
Glycyl-L-leucine	6.90E-04	1.10	16.07			
PC (O-18:4/0:0)	4.92E-02	1.25	4.43			
PC (20:5/0:0)	7.89E-04	1.19	7.88	6.65E-03	1.01	4.89
Azelaic acid	1.50E-02	1.14	34.99			
PG (18:1/18:1)	8.29E-08	2.16	3.66	3.13E-04	1.33	3.02
L-valine	8.06E-03	1.16	1.36			
4-hydroxybenzoate	1.58E-04	1.02	4.65			
D-allose	1.12E-06	2.37	1.82	1.65E-06	1.80	1.74
Alpha-D-glucose	6.83E-08	2.23	1.67	1.57E-07	1.54	1.67
L-arginine	8.44E-13	2.58	2.43	1.99E-10	1.89	2.06
L-histidine	1.60E-02	1.21	1.13	3.73E-05	1.54	1.30
LPS (20:2/0:0)				3.21E-04	1.34	2.21
PC (18:1/0:0)				4.98E-06	1.51	1.77
PC (18:2/0:0)				1.79E-03	1.19	1.36
PE (20:4/0:0)	2.61E-02	1.31	1.31	5.34E-04	1.11	1.58
LPS (18:3/0:0)	1.03E-03	1.64	1.43	4.51E-07	1.74	1.92
PE (18:2/0:0)	1.71E-02	1.31	1.35	6.51E-06	1.57	1.77
LPS (18:2/0:0)	2.81E-03	1.27	1.63	9.35E-07	1.71	2.93
PE (18:1/0:0)	2.11E-03	1.47	1.53	1.84E-05	1.34	1.81
PE (18:3/0:0)				1.35E-04	1.20	2.26
PA (20:3/0:0)				1.49E-04	1.25	1.25
PI (18:0/18:2)				2.86E-03	1.03	1.48

*VIP, variable importance in the projection; FC, fold change.*

*^a^P-values were derived from two-tailed Student’s t-test (subject to normal distribution) or Mann-Whitney U-test (not subject to normal distribution).*

### Pathway Analysis of Sex Differences in Altered Plasma Metabolites

**FIGURE 2:**
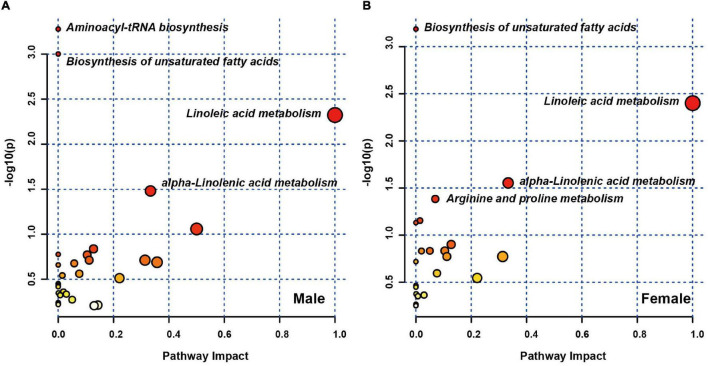
Pathway analysis of sex differences in differential plasma metabolites. Metabolic pathway analysis plot displaying the four affected metabolic pathways in male **(A)** and female **(B)** sets.

To further explore the biological functions of these differential metabolites in both male and female sets, MetaboAnalyst 5.0 was performed. The pathways that were considerably affected are listed in Supplementary Table 1. In the male set, there were four significantly altered pathways (*P*-value < 0.05), including aminoacyl-tRNA biosynthesis, biosynthesis of unsaturated fatty acids, linoleic acid metabolism and alpha-linolenic acid metabolism (Figure 2A). In the female set, there were also four significantly altered pathways, including biosynthesis of unsaturated fatty acids, linoleic acid metabolism, alpha-linolenic acid metabolism and arginine and proline metabolism (Figure 2B). Among the three shared pathways, in comparison to HCs, both male and female subjects with MDD exhibited lower levels of fatty acids (FAs), such as palmitic acid and polyunsaturated fatty acids (PUFAs), including linoleic acid, arachidonic acid, eicosapentaenoic acid, alpha-linolenic acid, and phosphatidylcholine (PC) (16:0/18:2). Male subjects with MDD showed lower levels of L-phenylalanine, L-methionine and L-tryptophan, and higher levels of L-histidine, L-arginine and L-valine, in aminoacyl-tRNA biosynthesis. Female subjects with MDD had lower levels of creatine and D-proline, and a higher level of L-arginine, in arginine and proline metabolism.

### Simplified Sex-Specific Metabolite Biomarker Panel for Diagnosis of Major Depressive Disorder

**FIGURE 3:**
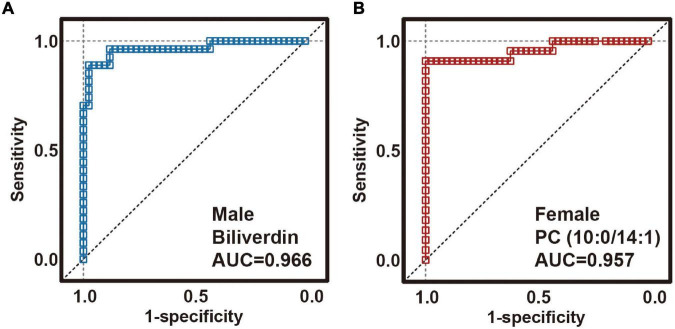
Diagnostic performance of sex-specific plasma biomarkers. Receiver operating characteristic (ROC) analysis showing outstanding diagnostic performances of these sex-specific biomarkers. **(A)** The area under the curve (AUC) of the male-specific biomarker was 0.966. **(B)** AUC of the female-specific biomarker was 0.966.

In clinical practice, measuring 57 or 53 plasma metabolites simultaneously to diagnose MDD is inconvenient and costly. Considering that a diagnostic panel based on the quantification of a small number of parameters would be more feasible and cost-effective, random forest analysis was used to simplify candidate metabolite biomarkers, and to describe the significant deviations between the MDD and HC samples. For a sex-specific metabolite biomarker, we selected a metabolite with the highest importance value (Supplementary Table 2). We found that a male-specific biomarker panel including biliverdin could effectively capture the significant divergence between MDD and HCs. Receiver operating characteristic analysis was performed to quantify the diagnostic performance with an area under the curve (AUC) of 0.966 (Figure 3A; 95% confidence interval: 0.921–1; sensitivity: 88.9%; specificity: 97.6%). A female-specific biomarker panel that included PC (10:0/14:1) was shown to be effective in capturing the significant difference between MDD and HC samples, with an AUC of 0.957 (Figure 3B; 95% confidence interval: 0.896–1; sensitivity: 90.9%; specificity: 100.0%). Because an AUC of 0.5 indicates a valueless test and an AUC of 1.0 indicates an excellent test, these results suggest that the two plasma metabolite panels are good classifiers of male and female subjects with MDD for both children and adolescents.

## Discussion

To the best of our knowledge, this is the first investigation of potential sex-specific biomarkers of MDD in children and adolescents through the metabolic profiling of plasma samples. Here, the coefficient loading plots of the OPLS-DA model identified 57 and 53 differential metabolites that can discriminate male and female patients with MDD, respectively, from HCs. Results of pathway analysis showed that there were four significantly altered pathways in both male and female patients with MDD, three of which were common to both male and female patients. Finally, this study identified two panels of plasma metabolite biomarkers, one with biliverdin for males and the other with PC (10:0/14:1) for females, allowing MDD and HC samples to be distinguished effectively with an AUC of 0.966 (male) and 0.957 (female). These two panels may aid in the development of a plasma-based diagnostic test for males and females with MDD in children and adolescents, as well as give new insight into the pathophysiology of MDD in both males and females.

Previous studies have found gender differences in lipid and glucose metabolism between males and females in healthy individuals (26). There is accumulating evidence suggesting that the contribution of long-chain polyunsaturated fatty acids to the fatty acid composition of plasma lipids may be higher in females than in males (27). As for patients with MDD, in adults, there were sex-specific modules in plasma mainly involved in bile acid biosynthesis and the carnitine shuttle for males and in glycosphingolipid metabolism and tryptophan metabolism for females; additionally, the porphyrin metabolism was a common pathway in both sexes (17). And in urine, significantly affected metabolic pathways mainly involved in pyrimidine metabolism and synthesis and degradation of ketone bodies for women and taurine and hypotaurine metabolism and aminoacyl-tRNA biosynthesis for men (28). Here, by comparing boys and girls with MDD with their respective HCs, we found that the types of fatty acids (FAs) that changed in the plasma of females with MDD were not identical to those of males with MDD. In comparison to the healthy population, the levels of the same fatty acids in female patients decreased more than male patients, although girls also had higher levels of long-chain polyunsaturated fatty acids in their plasma than boys. Compared with adult patients, altered metabolic pathways in boys and girls with MDD are very different, which deserves to be further studied.

By investigating the plasma metabolite signature of subjects with MDD, we found that the female MDD metabolic signatures were significantly different from that in male. The results showed that 17 metabolites were found to be notably changed in male patients. For example, the decrease in stearoylcarnitine was only shown in boys with MDD. Previous studies in male mice investigated the influence of stearoylcarnitine on energy metabolism in a hippocampal extracellular flux analyzer, which may lead to depression (29). Whereas 13 metabolites were found to be changed only in female patients. For instance, the decrease in creatine was only shown in girls with MDD. Females have been reported to have 70–80% lower endogenous creatine stores than males, and consume significantly lower amounts of dietary creatine compared to males (30). Dietary creatine supplementation has been shown to be beneficial for antidepressant treatment, possibly providing an energizing effect in brain chemistry through the efficient regeneration of intracellular high-energy phosphates in females (31, 32). And forty metabolites were significantly altered in both males and females with MDD. The physiopathological heterogeneity of MDD was reflected in the relatively diverse function of these altered metabolites. MetaboAnalyst 5.0 was performed to examine the metabolic pathways significantly affected by these differential metabolites (33). In both male and female sets, there were three significantly altered pathways consisting of the biosynthesis of unsaturated fatty acids, linoleic acid metabolism and alpha-linolenic acid metabolism. Compared with HCs, concentrations of FAs (e.g., cis-9-palmitoleic acid, dodecanoic acid and palmitic acid) were lower in children and adolescents with MDD, and these FAs determine membrane fluidity and peroxidizability as the major components of neuronal membranes (34). In depression, FA metabolism and its interaction with the hypothalamic-pituitary-adrenal axis are disrupted (35). Also, depression is associated with unique FA compositions in certain brain locations (36). Given these findings, together with a decrease in carnitine (10:0) that promotes fatty acids oxidation, we theorized that child–adolescent patients with MDD would experience a decline in fatty acid β-oxidation. In addition, a reduction of some polyunsaturated fatty acids [PUFAs, e.g., linoleic acid, arachidonic acid, eicosapentaenoic acid, alpha-linolenic acid, and PC (16:0/18:2)] has been found and reported in other study (37). The levels of PUFAs, adenosine, hypoxanthine and inosine were considerably lower in child–adolescent patients with MDD, demonstrating that PUFAs may play a role in the pathophysiology of depression through modulating the purine metabolism pathway (38). In the etiology of depression, depletion of omega-3 PUFAs has previously been linked to inflammatory processes and lipid peroxidation (39).

Although some biological functions are the same in both males and females with MDD (e.g., biosynthesis of unsaturated fatty acids), there are different changes in other pathways. For males, aminoacyl-tRNA biosynthesis (e.g., L-histidine, L-phenylalanine, L-arginine, L-methionine, L-valine, and L-tryptophan) was a significantly altered metabolic pathway in the current study. Perturbation of the aminoacyl-tRNA biosynthesis in peripheral blood appears to be prominently associated with patients exhibiting MDD (40–42). Depletion of L-phenylalanine and its derivative DL-O-tyrosine causes a reduction in dopamine levels, which is linked to the occurrence of depression (43). In humans, the reaction between L-methionine and adenosine triphosphate produces S-adenosyl-L-methionine, which has antidepressant effects, and a decrease in methionine would reduce its effect (44). Accumulation of kynurenine, an intermediate metabolite of tryptophan, in the brain is linked to the development of depression (45). For the female set, arginine and proline metabolism (e.g., L-arginine, creatine, and D-proline) were altered in the present study. It was reported that decreased arginine levels led to increased arginase activity, which affects the severity of depression (46). Arginine and proline metabolism is associated with the nitric oxide (NO) cycle (47). High level of NO in the brain was associated with elevated levels of interleukin-6 and tumor necrosis factor-α, both of which can contribute to cognitive impairment through tau phosphorylation (48). Thus, arginine and proline metabolism may have a role in MDD by mediating the inflammatory response.

Because of the following limitations, the findings should be regarded with caution. First, this work reanalyzed the data from our published study, which contained 133 child–adolescent participants, and only one metabolite was identified to distinguish MDD from HCs in male or female set. Follow up studies should expand the sample size, particularly the number of healthy controls, and apply targeted metabolomics to validate the diagnostic efficacy of the two biomarkers. Second, both drug-naive and drug-treated MDD participants were enrolled in this study, and female MDD participants showed higher CDRS-R score, which may have the potential to influence metabolomics results. Third, this work analyzed the metabolites in plasma; however, we neglected to record blood routine test, liver function, kidney function, and blood lipids despite the subjects’ self-reported absence of somatic diseases, which might not better explain the results. Fourth, clinical locations in a single city (Chongqing, China) were investigated in the current study, and dietary disparities (including the lower body mass index of individuals in China) between China and European nations may have an impact on metabolomic data (49, 50). Multiple centers are needed to validate the diagnostic efficacy of these metabolite biomarkers in further studies. Last, because of the diverse physicochemical properties and widely varying concentrations of metabolites, future research should employ more metabolomics platforms and methods.

## Conclusion

In conclusion, we identified different plasma metabolic profiles between male and female children and adolescents with MDD by a UPLC-Q-TOF/MS metabolomics approach. Seventeen metabolites were altered only in male patients with MDD, while 13 metabolites were altered only in female patients with MDD, and yet 40 metabolites changed in both. Four altered pathways were revealed by pathway analysis in male and female patients with MDD, three of which were shared. Sex-specific plasma metabolite biomarkers were identified for the diagnosis of MDD. Both male-specific (biliverdin) and female-specific [PC (10:0/14:1)] biomarkers were able to distinguish male and female subjects with MDD from their corresponding HCs. These results may serve as a valid diagnostic tool for male and female patients with MDD vs. healthy controls, and will facilitate future research into the different etiologies of MDD in male and female children and adolescents.

## Data Availability Statement

The datasets generated and/or analyzed during the current study are not publicly available because the datasets relate to other unpublished projects, but are available from the corresponding author on reasonable request.

## Ethics Statement

The studies involving human participants were reviewed and approved by Institutional Review Board of Children’s Hospital of Chongqing Medical University. Written informed consent to participate in this study was provided by the participants’ legal guardian/next of kin.

## Author Contributions

PX, XZ, YJ, and MQ designed the research. YJ, MQ, TT, XL, and YY performed the research. YJ, MQ, JW, HW, and YH analyzed the data. YJ, MQ, TT, XZ, and PX drafted the manuscript. All authors contributed to the article and approved the submitted version.

## Conflict of Interest

The authors declare that the research was conducted in the absence of any commercial or financial relationships that could be construed as a potential conflict of interest.

## Publisher’s Note

All claims expressed in this article are solely those of the authors and do not necessarily represent those of their affiliated organizations, or those of the publisher, the editors and the reviewers. Any product that may be evaluated in this article, or claim that may be made by its manufacturer, is not guaranteed or endorsed by the publisher.

## Footnotes

1
www.metaboanalyst.ca
