# Supplementary material for: Identification of Sex-Specific Plasma Biomarkers Using Metabolomics for Major Depressive Disorder in Children and Adolescents
Source: Front Psychiatry. 2022 Jul 14;13:929207. doi: 10.3389/fpsyt.2022.929207 (PMC9329558; doi:10.3389/fpsyt.2022.929207)
Supplement: Supplementary file 1 [file Table_1.DOCX]

Supplementary Material

# Supplementary Figures and Tables

## Supplementary Figures
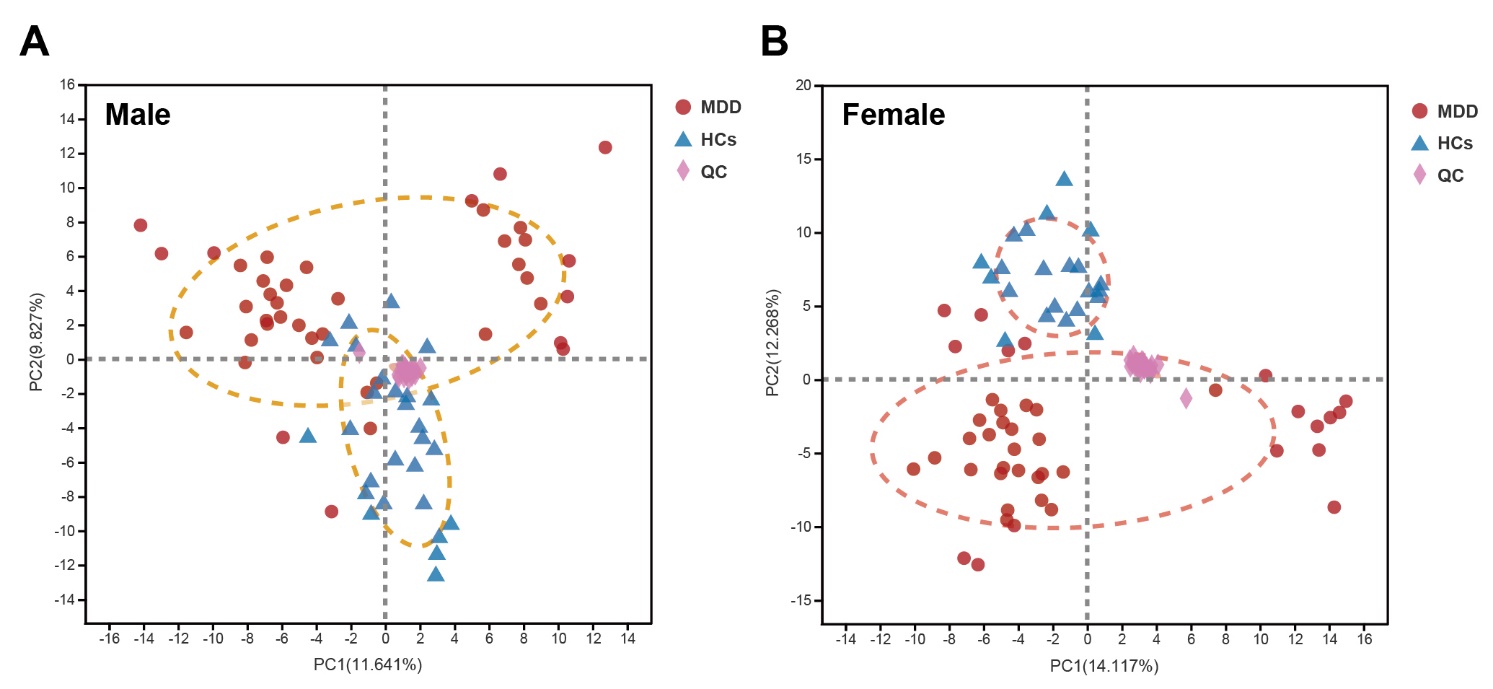
Supplementary Figure 1. Principal component analysis (PCA) score plots displaying distinct separations between male (A) and female (B) patients with major depressive disorder (MDD) from their respective healthy controls (HCs).

## Supplementary Tables

**Supplementary Table 1.** The correlation analysis between metabolites and CDRS-R scores.

| Metabolites | Male set | | Female set | |
| --- | --- | --- | --- | --- |
|  | r | p-value | r | p-value |
| PC(16:1/22:6) |  |  | 0.153 | 3.35E-01 |
| PA(22:5/0:0) | -0.089 | 5.74E-01 |  |  |
| PE(22:4/0:0) | 0.045 | 7.77E-01 |  |  |
| Adenosine |  |  | 0.133 | 4.02E-01 |
| PC(20:4/0:0) | 0.299 | 5.42E-02 | 0.158 | 3.19E-01 |
| PC(22:6/0:0) |  |  | 0.032 | 8.40E-01 |
| Biliverdin | 0.386 | 1.16E-02 | -0.087 | 5.85E-01 |
| L-Palmitoylcarnitine | 0.222 | 1.58E-01 | 0.038 | 8.09E-01 |
| 3-Methylthiopropionate | 0.350 | 2.30E-02 | 0.144 | 3.62E-01 |
| Stearoylcarnitine | 0.625 | 9.50E-06 |  |  |
| carnitine(18:1) | 0.286 | 6.64E-02 | 0.123 | 4.40E-01 |
| L-Phenylalanine | 0.211 | 1.80E-01 |  |  |
| L-Tryptophan | 0.547 | 1.80E-04 |  |  |
| DL-O-tyrosine | 0.576 | 6.52E-05 |  |  |
| PC(22:5/0:0) | 0.515 | 4.79E-04 | 0.258 | 9.93E-02 |
| PC(20:3/0:0) | 0.473 | 1.57E-03 |  |  |
| Acetylcarnitine | -0.481 | 1.25E-03 | 0.247 | 1.14E-01 |
| PC(12:0/22:5) | -0.584 | 4.99E-05 | -0.316 | 4.17E-02 |
| PC(20:5/24:4) | -0.594 | 3.37E-05 | -0.271 | 8.30E-02 |
| PC(10:0/14:1) | -0.618 | 1.28E-05 | -0.202 | 2.00E-01 |
| Bilirubin | -0.033 | 8.34E-01 | -0.028 | 8.62E-01 |
| 1-Methyladenosine | -0.491 | 9.61E-04 | -0.173 | 2.74E-01 |
| Linoleic acid | 0.073 | 6.46E-01 | -0.096 | 5.47E-01 |
| alpha-Linolenic acid | 0.061 | 7.00E-01 | -0.117 | 4.61E-01 |
| cis-9-Palmitoleic acid | -0.070 | 6.62E-01 | -0.116 | 4.64E-01 |
| trans-Vaccenic acid | -0.022 | 8.89E-01 | -0.086 | 5.87E-01 |
| Palmitic acid | -0.108 | 4.98E-01 | -0.068 | 6.69E-01 |
| Arachidonic Acid | -0.296 | 5.73E-02 | -0.133 | 4.00E-01 |
| Eicosapentaenoic acid | -0.112 | 4.82E-01 | -0.066 | 6.76E-01 |
| Capric acid |  |  | -0.094 | 5.53E-01 |
| carnitine(10:0) |  |  | -0.063 | 6.91E-01 |
| Dodecanoic acid | -0.200 | 2.05E-01 | -0.080 | 6.14E-01 |
| PC(16:0/18:2) | 0.300 | 5.32E-02 | 0.231 | 1.41E-01 |
| L-Methionine | -0.471 | 1.66E-03 | -0.295 | 5.82E-02 |
| Indole | 0.203 | 1.97E-01 |  |  |
| Indoleacrylic acid | 0.177 | 2.62E-01 |  |  |
| Hypoxanthine | -0.540 | 2.22E-04 | -0.176 | 2.66E-01 |
| Inosine | -0.575 | 6.79E-05 | -0.050 | 7.51E-01 |
| Dopamine | -0.575 | 6.83E-05 |  |  |
| Betaine | -0.573 | 7.26E-05 | -0.413 | 6.54E-03 |
| L-Carnitine | -0.475 | 1.48E-03 | -0.161 | 3.08E-01 |
| Creatine |  |  | -0.196 | 2.13E-01 |
| D-Proline | -0.291 | 6.18E-02 | -0.284 | 6.85E-02 |
| Creatinine | -0.621 | 1.14E-05 | -0.386 | 1.15E-02 |
| L-Pyroglutamic acid | -0.192 | 2.23E-01 | -0.251 | 1.08E-01 |
| PG(16:0/0:0) | -0.356 | 2.07E-02 |  |  |
| PC(13:0/22:2) | -0.676 | 8.88E-07 |  |  |
| PC(18:2/24:4) |  |  | -0.227 | 1.49E-01 |
| Glycyl-L-leucine | -0.499 | 7.69E-04 |  |  |
| PC(O-18:4/0:0) | -0.675 | 9.37E-07 |  |  |
| PC(20:5/0:0) | -0.458 | 2.31E-03 | -0.204 | 1.95E-01 |
| Azelaic acid | -0.631 | 7.38E-06 |  |  |
| PG(18:1/18:1) | -0.539 | 2.27E-04 | -0.141 | 3.73E-01 |
| L-Valine | 0.046 | 7.71E-01 |  |  |
| 4-Hydroxybenzoate | -0.039 | 8.05E-01 |  |  |
| D-Allose | 0.596 | 3.13E-05 | 0.237 | 1.30E-01 |
| Alpha-D-Glucose | 0.493 | 9.17E-04 | 0.256 | 1.01E-01 |
| L-Arginine | 0.285 | 6.76E-02 | 0.063 | 6.92E-01 |
| L-Histidine | 0.292 | 6.03E-02 | 0.012 | 9.40E-01 |
| LPS(20:2/0:0) |  |  | -0.135 | 3.92E-01 |
| PC(18:1/0:0) |  |  | -0.082 | 6.06E-01 |
| PC(18:2/0:0) |  |  | -0.025 | 8.73E-01 |
| PE(20:4/0:0) | 0.145 | 3.60E-01 | 0.109 | 4.94E-01 |
| LPS(18:3/0:0) | 0.134 | 3.97E-01 | 0.239 | 1.27E-01 |
| PE(18:2/0:0) | 0.238 | 1.28E-01 | 0.308 | 4.75E-02 |
| LPS(18:2/0:0) | 0.060 | 7.07E-01 | 0.225 | 1.52E-01 |
| PE(18:1/0:0) | 0.080 | 6.13E-01 | 0.153 | 3.33E-01 |
| PE(18:3/0:0) |  |  | 0.167 | 2.90E-01 |
| PA(20:3/0:0) |  |  | 0.245 | 1.18E-01 |
| PI(18:0/18:2) |  |  | 0.214 | 1.73E-01 |

**Supplementary Table 2.** The significantly altered pathways in both male and female sets.

| Male set | | | Female set | | |
| --- | --- | --- | --- | --- | --- |
| Pathway | p-value | Impact | Pathway | p-value | Impact |
| Aminoacyl-tRNA biosynthesis | 5.27E-04 | 0 | Biosynthesis of unsaturated fatty acids | 6.50E-04 | 0 |
| Biosynthesis of unsaturated fatty acids | 9.96E-04 | 0 | Linoleic acid metabolism | 3.97E-03 | 1 |
| Linoleic acid metabolism | 4.75E-03 | 1 | alpha-Linolenic acid metabolism | 2.80E-02 | 0.33333 |
| alpha-Linolenic acid metabolism | 3.31E-02 | 0.33333 | Arginine and proline metabolism | 4.13E-02 | 0.06998 |

**Supplementary Table 3.** The importance of differentiating metabolites in both male and female random forest models.

| Male set | | Female set | | | |  |  |
| --- | --- | --- | --- | --- | --- | --- | --- |
| Metabolites | Importance | | Metabolites | | Importance | | |
| Biliverdin | 10.002 | | PC(10:0/14:1) | | 8.849 | | |
| Inosine | 9.660 | | PC(12:0/22:5) | | 8.601 | | |
| L-Arginine | 8.182 | | Inosine | | 8.512 | | |
| Hypoxanthine | 8.049 | | PC(20:5/24:4) | | 8.326 | | |
| PC(12:0/22:5) | 7.510 | | Adenosine | | 8.157 | | |
| PG(18:1/18:1) | 7.290 | | Biliverdin | | 6.841 | | |
| PC(10:0/14:1) | 7.138 | | Bilirubin | | 6.723 | | |
| PC(20:5/24:4) | 7.125 | | L-Arginine | | 5.672 | | |
| Alpha-D-Glucose | 5.437 | | PC(22:6/0:0) | | 4.913 | | |
| D-Allose | 4.995 | | Creatinine | | 4.698 | | |
| L-Tryptophan | 4.986 | | D-Allose | | 4.451 | | |
| Azelaic acid | 4.964 | | cis-9-Palmitoleic acid | | 4.242 | | |
| Creatinine | 4.813 | | 1-Methyladenosine | | 4.206 | | |
| PC(20:5/0:0) | 4.543 | | Betaine | | 3.928 | | |
| Betaine | 4.538 | | Hypoxanthine | | 3.861 | | |
| PC(O-18:4/0:0) | 4.476 | | trans-Vaccenic acid | | 3.611 | | |
| L-Methionine | 4.230 | | Acetylcarnitine | | 3.536 | | |
| PC(13:0/22:2) | 4.183 | | LPS(18:3/0:0) | | 3.520 | | |
| Acetylcarnitine | 4.169 | | PC(18:1/0:0) | | 3.463 | | |
| DL-O-tyrosine | 4.044 | | Palmitic acid | | 3.392 | | |
| PC(22:5/0:0) | 3.968 | | Alpha-D-Glucose | | 3.388 | | |
| Glycyl-L-leucine | 3.689 | | alpha-Linolenic acid | | 2.916 | | |
| L-Pyroglutamic acid | 3.336 | | Linoleic acid | | 2.907 | | |
| carnitine(18:1) | 3.169 | | carnitine(18:1) | | 2.628 | | |
| Bilirubin | 3.160 | | LPS(18:2/0:0) | | 2.530 | | |
| L-Carnitine | 2.969 | | PE(18:2/0:0) | | 2.492 | | |
| PC(16:0/18:2) | 2.945 | | Creatine | | 2.300 | | |
| Dopamine | 2.753 | | PC(20:4/0:0) | | 2.293 | | |
| Arachidonic Acid | 2.263 | | PC(18:2/0:0) | | 2.282 | | |
| Eicosapentaenoic acid | 2.070 | | L-Carnitine | | 2.222 | | |
| LPS(18:3/0:0) | 1.983 | | Capric acid | | 2.156 | | |
| L-Palmitoylcarnitine | 1.941 | | carnitine(10:0) | | 2.030 | | |
| alpha-Linolenic acid | 1.898 | | PC(22:5/0:0) | | 2.014 | | |
| PE(18:2/0:0) | 1.896 | | L-Pyroglutamic acid | | 1.919 | | |
| L-Valine | 1.722 | | L-Methionine | | 1.907 | | |
| Stearoylcarnitine | 1.594 | | L-Palmitoylcarnitine | | 1.882 | | |
| D-Proline | 1.593 | | LPS(20:2/0:0) | | 1.869 | | |
| Indole | 1.458 | | PC(20:5/0:0) | | 1.866 | | |
| PE(22:4/0:0) | 1.424 | | PE(18:3/0:0) | | 1.632 | | |
| PE(20:4/0:0) | 1.414 | | L-Histidine | | 1.613 | | |
| PE(18:1/0:0) | 1.299 | | PE(20:4/0:0) | | 1.609 | | |
| Dodecanoic acid | 1.248 | | Eicosapentaenoic acid | | 1.608 | | |
| 4-Hydroxybenzoate | 1.220 | | PC(16:0/18:2) | | 1.566 | | |
| L-Phenylalanine | 1.115 | | 3-Methylthiopropionate | | 1.544 | | |
| 3-Methylthiopropionate | 1.105 | | PG(18:1/18:1) | | 1.344 | | |
| 1-Methyladenosine | 1.007 | | PC(18:2/24:4) | | 1.151 | | |
| PC(20:4/0:0) | 0.997 | | PA(20:3/0:0) | | 1.148 | | |
| PC(20:3/0:0) | 0.701 | | PI(18:0/18:2) | | 0.668 | | |
| cis-9-Palmitoleic acid | 0.360 | | D-Proline | | 0.614 | | |
| Palmitic acid | 0.350 | | PE(18:1/0:0) | | 0.431 | | |
| Indoleacrylic acid | 0.179 | | Dodecanoic acid | | -0.098 | | |
| PG(16:0/0:0) | 0.175 | | Arachidonic Acid | | -0.358 | | |
| L-Histidine | -0.061 | | PC(16:1/22:6) | | -1.417 | | |
| trans-Vaccenic acid | -0.318 | |  | |  | | |
| PA(22:5/0:0) | -0.583 | |  |  | | |  |
| LPS(18:2/0:0) | -0.615 | |  |  | | |  |
| Linoleic acid | -1.390 | |  |  | | |  |
